# Current Radiotherapy Management of Extensive-Stage Small-Cell Lung Cancer in the Immunotherapy Era: An Italian National Survey on Behalf of the Italian Association of Radiotherapy and Clinical Oncology (AIRO)

**DOI:** 10.3390/curroncol31110501

**Published:** 2024-11-01

**Authors:** Alessio Bruni, Vieri Scotti, Maria Alessia Zerella, Federica Bertolini, Jessica Imbrescia, Emanuela Olmetto, Chiara Bennati, Francesco Cuccia, Marianna Miele, Niccolò Giaj-Levra, Marcello Tiseo, Patrizia Ciammella, Stefano Vagge, Marco Galaverni, Antonio Pontoriero, Serena Badellino, Ruggero Spoto, Emanuele Alì, Paolo Borghetti

**Affiliations:** 1 Radiation Oncology Unit, Department of Oncology and Hematology, University Hospital of Modena, 41124 Modena, Italy; bruni.alessio@aou.mo.it (A.B.);; 2 Radiation Oncology Unit, Oncology Department, Azienda Ospedaliero Universitaria Careggi, 50134 Firenze, Italy; 3 Division of Radiotherapy, European Institute of Oncology, Istituto di Ricovero e Cura a Carattere Scientifico, 20141 Milano, Italy; 4 Division of Oncology, Department of Oncology and Hematology, Modena University Hospital, 41124 Modena, Italy; 5 Department of Onco-Hematology, AUSL della Romagna, 48121 Ravenna, Italy; 6 Radiotherapy Unit, ARNAS Civico Hospital, 90145 Palermo, Italy; 7 Operative Research Unit of Radiation Oncology, Fondazione Policlinico Universitario Campus Bio-Medico, 00128 Rome, Italy; 8 Advanced Radiation Oncology Department, Cancer Care Center, IRCCS Sacro Cuore Don Calabria Hospital, 37024 Negrar, Italy; 9 Department of Medicine and Surgery, University of Parma, 43126 Parma, Italy; 10 Medical Oncology Unit, University Hospital of Parma, 43126 Parma, Italy; 11 Radiation Oncology Unit, Azienda USL-Istituto di Ricovero e Cura a Carattere Scientifico Di Reggio Emilia, 42123 Reggio Emilia, Italy; 12 Radiotherapy Department, E.O. Ospedali Galliera, 16128 Genova, Italy; 13 Radiation Oncology Unit, University Hospital of Parma, 43126 Parma, Italy; 14 Radiation Oncology Unit, Department of Biomedical, Dental and Morphological and Functional Imaging Sciences, University of Messina, 98122 Messina, Italy; 15 Radiation Oncology, Department of Oncology, University of Turin, 10125 Turin, Italy; 16 Department of Radiotherapy and Radiosurgery, Istituto di Ricovero e Cura a Carattere Scientifico Humanitas Research Hospital, 20089 Milan, Italy; 17 Radiation Oncology Department, ASST Spedali Civili and University of Brescia, P.le Spedali Civili, 1, 25123 Brescia, Italy; paolo.borghetti@asst-spedalicivili.it

**Keywords:** radiation therapy, small-cell lung cancer, national Italian survey

## Abstract

Background: Extensive-stage small-cell lung cancer (ES-SCLC) treatment has recently been revolutionized by the advent of immune checkpoint inhibitors. This survey was conducted to evaluate the current pattern of care among Italian clinicians, in particular about the integration with radiation therapy (RT). Methods: In June 2023, 225 Italian cancer care professionals were invited to complete a 21-question web-based survey about ES-SCLC management through personal contacts and the Italian Association for Radiotherapy and Clinical Oncology (AIRO) network. Results: We received 90 responses; the majority were radiation oncologists (89%) with more than 10 years of experience (51%). The preferred management of ES-SCLC in patients with a good performance status was concomitant chemo-immunotherapy (84%). Almost all respondents recommended prophylactic cranial irradiation (PCI) (85%), taking into account age and thoracic response; PCI was performed mainly between the end of chemotherapy and before starting immunotherapy (37%), with a three-dimensional conformal technique (46%). Furthermore, 83% of respondents choose to deliver thoracic RT in the case of both an intrathoracic and extrathoracic response, with an RT schedule of 30 Gy/10 fractions. Stereotactic RT is increasingly being used in oligoprogressions. Conclusions: Our analysis showed the variability of real-world management of ES-SCLC. Future clinical trials and developments are needed to improve the multidisciplinary treatment of these patients.

## 1. Introduction

Small-cell lung cancer (SCLC) is a really aggressive and poorly differentiated malignancy, and approximately only one third of patients are diagnosed with localized or locoregional disease. SCLC accounts for about 13–15% of new diagnoses of lung cancers, and it represents the second most common thoracic malignancy [1]. Its incidence is strongly related to a smoking habit, and only 2% of SCLC cases arise in never smokers. Within this subgroup of patients, some cases derive from the histological transformation of oncogene-addicted (EGFR- or ALK-driven) lung adenocarcinoma to SCLC [2].

SCLC is usually staged according to the Veterans Administration Lung Study Group (VALG) staging system, which defines the limited stage (LS-SCLC) as the disease confined to one hemithorax, potentially being treated with radical radiotherapy (corresponding to stage I–III of the 8th edition of the American Joint Committee on Cancer—AJCC), and the extensive stage (ES-SCLC) corresponding to stage IVA/B of the 8th edition AJCC [3].

LS-SCLC is potentially curable, and actually the standard of care is concurrent chemoradiotherapy, with a median overall survival (OS) of about 25–30 months and a 5-year OS rate of 20–30%, followed by prophylactic cranial irradiation (PCI) [1,4]. Furthermore the ADRIATIC trial recently showed that immunotherapy with the immune checkpoint inhibitor (ICI) durvalumab, targeting programmed death-ligand 1 (PD-L1), led to a significantly longer OS and progression-free survival (PFS) than placebo, building a potential new approach to care in patients with LS-SCLC [5].

On the other hand, the prognosis for the extensive stage (ES-SCLC) has remained poor over the past 20 years, with a median OS of about 10 months and a 5-year OS rate < 5%. The treatment was historically based on six cycles of chemotherapy with cisplatin or carboplatin plus etoposide; PCI and consolidative thoracic RT (ctRT) were offered to patients with a tumor response after the initial systemic treatment to control local disease and improve the OS [6,7].

Recently, two phase III randomized clinical trials (RCTs) have changed the treatment paradigm of ES-SCLC. These trials showed the benefit of adding durvalumab or atezolizumab, another ICI targeting PD-L1, to platinum-based chemotherapy, followed by maintenance with ICIs alone, leading to an OS and PFS benefit [8,9,10].

However, both trials did not include ctRT, for which robust evidence on safety, efficacy, and optimal timing in the chemo-immunotherapy era is not yet available. The results from several early-phase and retrospective analyses suggest improved clinical outcome and good toxicity profiles of ctRT such that the American Society for Radiation Oncology (ASTRO) guidelines and a more recent Canadian consensus recommended ctRT in patients responding to first-line chemo-immunotherapy and having a good performance status (PS). Even if these findings seem to be very promising, actually we still need more robust data, so that randomized studies are currently ongoing [11,12,13,14].

PCI is another highly-debated topic in SCLC management, considering that among patients who undergo chemo-radiotherapy, approximately 59% to 69% have a significant risk of developing brain metastases. PCI is recommended in patients responding to systemic therapy and having a good PS; however Takayasu et al. recently highlighted the value of MRI surveillance, showing no survival benefit of PCI compared with observation in patients with ES-SCLC [10]. These results are likely to have influenced clinical practice, given the potential impact of PCI on quality of life, even if more data are needed to confirm these insights [1,15]. Additionally, the CASPIAN trial [8,9] and IMPOWER133 trial [10] resulted in a low adoption of PCI, making more challenging the role of PCI in extensive disease in this era of immunotherapy.

Many questions still remain open, such as the optimal timing and dose of ctRT in the era of chemo-immunotherapy in ES-SCLC, the best management of patients with a poor performance status or the use of PCI and its optimal timing. So, on behalf of the Italian Association of Radiotherapy and Clinical Oncology (AIRO), this survey was designed to evaluate the current management of ES-SCLC in Italy in order to identify differences in SCLC care practices and consequently to optimize patients’ treatment.

## 2. Materials and Methods

Beginning some years ago with a first survey on the management of SCLC [16], the thoracic oncology AIRO study group planned a 21-question web-based survey about ES-SCLC management; the questions posed to respondents are reported in the Supplementary Material (Table S1).

The survey was planned in November 2022 in light of a national event that involved many different specialists (pneumologists, medical and radiation oncologists, radiologists). After obtaining the endorsement of the AIRO scientific committee, the final version was finalized and sent out by e-mail for the first time in June 2023, with a link to the online questionnaire, to the heads of all Italian radiation oncology departments or to the radiation oncologists’ deputies for lung cancer disease (205) and to the cancer care professionals involved in the national event (5 pneumologists, 12 medical oncologists, 3 other cancer care professionals). A second call was sent out two months later to maximize the response rate. A newsletter from the thoracic oncology AIRO study group and a list of Italian radiation departments’ chiefs was used to get in touch with as many specialists as possible.

The survey was conducted anonymously, and it was structured in two parts: the first one concerned demographic questions about the physicians’ experience in lung cancer management, the number of patients with SCLC treated annually in each oncological center and role of multidisciplinary discussion in the clinical decision-making process. Conversely, the second part of the survey was dedicated to the specific management of patients affected by ES-SCLC.

Physicians were asked to select the answers that most closely matched their standard of care about the following: the use of immunotherapy in association with a standard chemotherapy regimen in both fit and frail patients, the combination of ctRT with chemo-immunotherapy, focusing on its timing and fractionation, the factors influencing the use of PCI and types of RT planning techniques and delivery. It was developed using a survey-monkey web link, and all answers were deemed eligible for statistical analysis.

The study was approved by the scientific committee of AIRO (Nr. 6/2022).

## 3. Results

### 3.1. Demographics and Expertise in ES-SCLC Treatment

A total of 90 out of 225 Italian cancer care professionals answered the survey (40% response rate); their characteristics are listed in Table 1.

The majority of respondents were radiation oncologists (89%), followed by medical oncologists (6%), pneumologists (94%) and other specialties (1%); however, 69% of them spent more than 50% of the time working within the scope of lung cancer disease.

Fifty-one per cent of respondents had more than 10 years of experience; 46% worked in non-academic hospitals; almost all the other half worked in academic hospitals or cancer care centers (48%).

**Figure 1:**
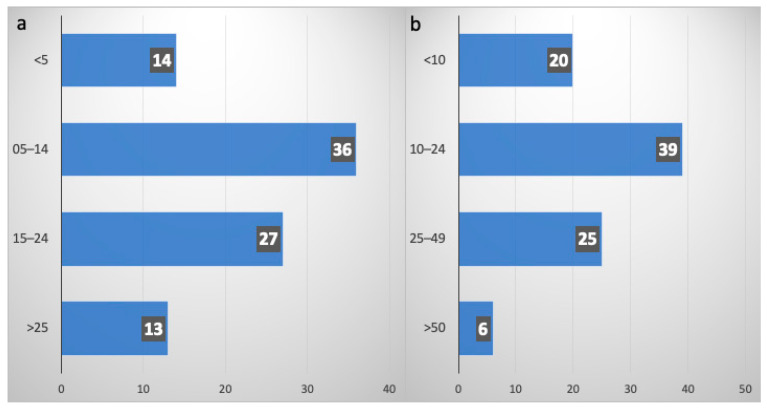
(**a**) Numbers of newly diagnosed SCLC patients per year; (**b**) mewly diagnosed ES-SCLC patients per year (**b**). On the vertical axes, the number of new diagnoses collected for subgroups in increasing order; on the horizontal axes, the absolute numbers of new diagnoses in ascending order.

Weekly MDT discussions for SCLC cases were reported by 79% of respondents; the specialists more commonly involved in diagnosis and staging were clinical oncologists (46%) and pneumologists (39%). The number of newly diagnosed SCLC patients seen annually was >50 only in 7% of cases, less than 10 in 22% and between 10 and 49 for over a half (71%), as shown in Figure 1a. Specifically, for newly diagnosed ES-SCLC, the majority of the respondents (70%) treated between 5 and 24 patients/year (Figure 1b).

### 3.2. Management of ES-SCLC

#### 3.2.1. Role of Systemic Treatment

Regarding patients with ES-SCLC and PS = 0–1, 84% of respondents declared that their first therapeutic approach was concomitant chemo-immunotherapy; specifically, 66% of them preferred to start both concomitantly from the first cycle, while 18% of them preferred it from the second or third cycle of chemotherapy.

When choosing a chemotherapy regimen for ES-SCLC, the majority of the respondents preferred carboplatin + etoposide + atezolizumab both for PS 0–1 (36%) and PS 2 patients (32%); as a second option, cisplatin + etoposide +/− durvalumab was administered to patients with PS = 0–1, but usually it was not chosen for PS 2 ones.

Interestingly, no responders declared precluding an immunotherapy-based treatment in patients with PS 0–1, and only 6% did not offer immunotherapy in patients with PS 2; four cycles of chemotherapy were the preferred option for 68% of clinicians if a combination of chemotherapy and immunotherapy was offered.

#### 3.2.2. Role of PCI

**Figure 2:**
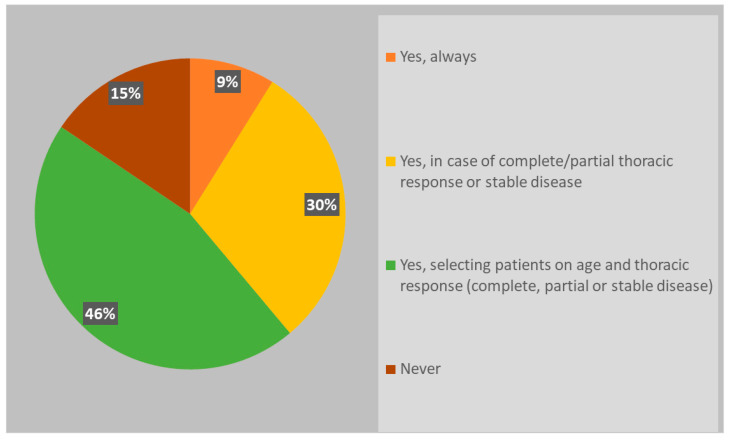
PCI recommendations in patients with ES-SCLC.

**Figure 3:**
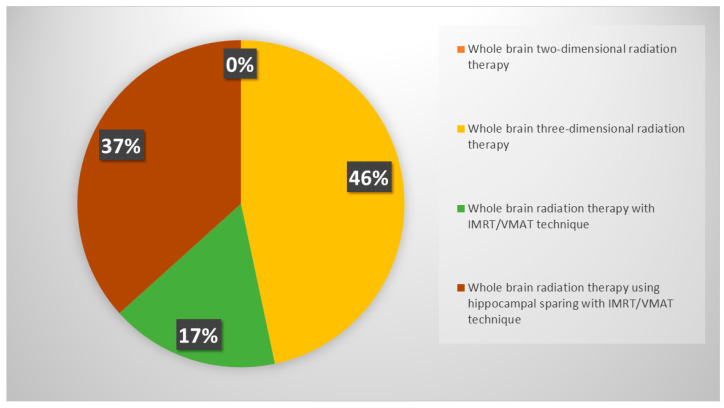
PCI technique of choice according to respondents.

Although the new scenario related to the wide adoption of immunotherapy in ES-SCLC, almost all radiation oncologists (85%) usually recommend PCI (Figure 2); respondents were keener to perform PCI in patients selected on age and thoracic response (complete, partial or stable disease) (46%), while 30% of respondents declared considering only thoracic response. PCI was never offered in an ES-SCLC setting by 15% of clinicians. If prescribed, PCI is mostly performed after the end of chemo-immunotherapy, before starting the first cycle of maintenance immunotherapy (37%) or in concomitance with it (22%); several respondents declared waiting until the completion of the first cycle of immunotherapy, both discontinuing immunotherapy during PCI (24%) and not (17%). Whole brain three-dimensional conformal radiation therapy (3DCRT) was used in the case of PCI by 46% of respondents, while intensity-modulated RT (IMRT)/volumetric modulated arc therapy (VMAT) with or without hippocampal avoidance was chosen by 37% and 17% of responders, respectively (Figure 3).

#### 3.2.3. Role of Thoracic Consolidative RT

**Figure 4:**
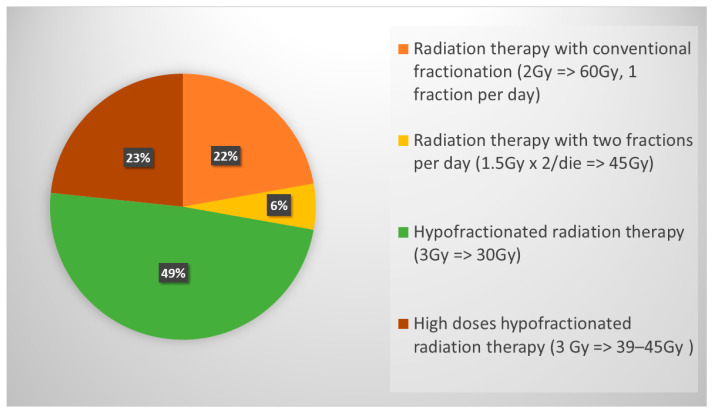
Schedules usually chosen for thoracic RT in ES-SCLC patients.

The role of ctRT in ES-SCLC was investigated in this survey, resulting in data that 83% of respondents choose to deliver RT in the case of both an intrathoracic and extrathoracic response (complete, partial or stable disease); a minority opted for RT considering only extrathoracic response (10%) and 7% declared considering it regardless of response. The RT schedule was 30 Gy in 10 daily fractions for the majority of respondents (49%) (Figure 4).

**Figure 5:**
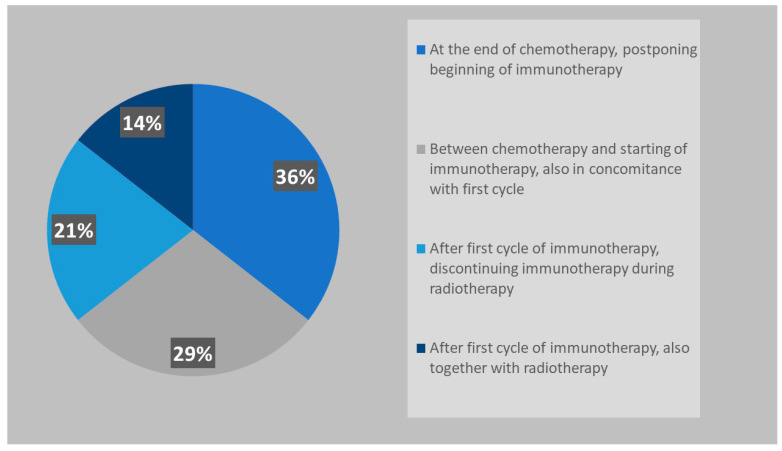
Timing of thoracic RT in patients with ES-SCLC.

Similarly to PCI, ctRT is also mostly performed between the end of chemotherapy and the beginning of immunotherapy (65%) (Figure 5).

#### 3.2.4. Management of Oligoprogression

If an intracranial oligoprogression occurred during maintenance immunotherapy, the treatment of choice was stereotactic RT (SRT) for 43% of clinicians, regardless of previous PCI. Conversely, if patients were RT-naive, 39% opted for WBRT. On the other hand, if extracranial oligoprogression occurred during maintenance immunotherapy, 67% of responders chose continuing the current systemic treatment, adding local treatment (SRT or palliative radiotherapy).

## 4. Discussion

The results of this survey, driven by the radiation oncology community, showed the variability of the real-world management of ES-SCLC after the introduction of first-line immunotherapy. The CASPIAN and the IMpower 133 randomized clinical trials demonstrated that the addition of durvalumab or atezolizumab to first-line chemotherapy improved the OS HR: 0.73 (95% CI 0.59–0.91; *p* = 0.0047) and 0.70 (95% CI 0.54–0.91; *p* = 0.007), respectively [8,9,10], if compared to chemotherapy alone. In both trials, patients received four cycles of chemotherapy in association with immunotherapy, followed by maintenance immunotherapy without a significant increase in toxicities. Our survey demonstrated that only 67% of clinicians strictly follow the CASPIAN or IMpower 133 schedules. Indeed, 18% of respondents prescribe immunotherapy from the second or third cycle of chemotherapy, 8% only after chemotherapy and 8% do not prescribe immunotherapy at all (commonly due to the frailty of these patients). The majority of clinicians usually perform four cycles of chemotherapy concomitant to immunotherapy; these results could be due to the recent introduction in clinical practice of these new regimens, which are counterintuitive if compared to the previous standard of care, in which six cycles of chemotherapy were provided to obtain the most chemotherapy efficacy. Furthermore, another reason for this discrepancy from the guidelines may be a consequence of not being fully dedicated to pulmonary pathology, as reported by almost 30% of respondents. According to our survey, 36% of clinicians preferred atezolizumab instead of durvalumab (25%), while for 24% of the participants both treatments were comparable. This is probably due to the fact that in Italy atezolizumab was refundable from 2020, durvalumab from 2022, and clinicians feel more confident in the use of one regimen rather than another. In addition, the fact that atezolizumab administration was made with carboplatin, which is commonly considered more manageable and less toxic, particularly in frail patients, should be considered. On the other hand, even if the use of cisplatin was allowed in the CASPIAN trial for fit patients, only a quarter of the population in both the durvalumab and placebo arm received it. Our findings revealed that the majority of clinicians still prefer to use carboplatin instead of cisplatin, probably because most patients were considered cisplatin-unfit. Both the CASPIAN and IMpower 133 trials enrolled patients with an ECOG PS 0–1, so no data are available for ECOG PS 2 patients. Agarwal et al. recently compared the use of immunotherapy regimens in patients affected by ES-SCLC with ECOG PS 0–1 vs. ECOG PS 2–3 patients without finding any difference in terms of both PFS and OS [17]. Many studies have shown that in real-world practice, ES-SCLC patients with ECOG PS 2 treated as per the CASPIAN or IMpower 133 regimens did not present increased toxicities, without any differences in terms of efficacy [18,19]. Similarly, our results showed that only 9% of clinicians proposed chemotherapy alone to these patients, while the vast majority use a combination of chemo and immunotherapy, and 6% even use a cisplatin combination.

Most Italian radiation oncologists (about 46%) would offer PCI only to patients with complete or partial remission after the first chemo-immunotherapy phase of treatment, considering the patient’s age and discouraging PCI in older patients with many comorbidities. On the contrary, 16% of respondents would never offer PCI in ES-SCLC. As we know, PCI was allowed during maintenance treatment only in the IMpower 133 trial, and it was offered to just 10% of patients in both the immunotherapy and placebo groups. In the pre-immunotherapy era, the phase III EORTC trial compared PCI to observation in ES-SCLC, with any response to first-line chemotherapy being demonstrated to increase the DFS HR: 0.76 (95% CI 0.59–0.96; *p* = 0.02) and to improve the OS HR: 0.68 (95% CI 0.52–0.88; *p* = 0.003) and 1-year survival rate 27.1% (95% CI 19.4 to 35.5) vs. 13.3% (95% CI 8.1 to 19.9) of the irradiation group compared to the observation group [20]. This trial was heavily criticized, as pre-PCI brain imaging was not required, and patients in the PCI group presented a lower risk of brain metastases development (14% compared to 40.4% of the control group), and long-term neurocognitive deterioration issues arose [21]. Opposite results were obtained from a phase III Japanese trial, where the authors demonstrated a detrimental effect of PCI compared to observation, which consisted of brain MRI every three months for the first 12 months and every 6 months thereafter [15].

Little evidence is available in the immunotherapy era. At ASTRO 2020, Higgings et al. reported the results of an exploratory analysis about the pattern of disease progression in patients enrolled in the Impower 133 trial. They observed an improved intra-cranial PFS in those patients who underwent PCI both in the atezolizumab and the placebo arm, even though only 22 (10%) of them underwent PCI in the two groups. The median time to intra-cranial progression was 20.2 months for atezolizumab and 10.5 months for placebo in the PCI group, and 16.5 months for atezolizumab and 9.8 months for placebo in the group without PCI, confirming the positive role of PCI in delaying the appearance of brain metastases [22].

Similarly, Chen et al. reported a median time to RECIST-defined disease progression in the brain or brain radiotherapy (excluding PCI), whichever occurred first; 19.2 months and 10.2 months for durvalumab plus chemotherapy and chemotherapy alone, respectively. In the CASPIAN trial, PCI was allowed only for the placebo group and applied in 8% of patients [23].

Gross et al. performed a study to evaluate the impact of PCI and thoracic radiotherapy after the introduction of immunotherapy. Considering all the limits of a monocentric non-randomized study, they observed in 54 patients who underwent PCI an improved OS from 10 to 15 months and an improved median PFS from 5 to 8.5 months [24].

The respondents in favor of PCI prefer to perform it before the first cycle of maintenance immunotherapy (37%) or even in association with it (39%); only 24% prefer to discontinue immunotherapy during PCI. Forty-seven percent of radiation oncologists use a 3D technique, 17% use IMRT, and 37% are in favor of hippocampal-sparing brain RT as it demonstrates a significant reduction in the risk of neurocognitive impairments [25]. Unfortunately, the role of PCI in ES-SCLC is still unclear, and the results of ongoing trials such as the MAVERIK and PRIMAlung studies are still awaited in order to find an answer to this important issue [26,27].

Before the CASPIAN and IMpower 133 publications, ctRT for ES-SCLC, achieving a clinical and radiological response to chemotherapy, was part of the management of these patients, and it is still suggested by American and European guidelines [28,29]. The RTOG 0937 trial randomized patients with ES-SCLC with any response to chemotherapy to receive PCI vs. PCI plus cRT (45 Gy in 15 fractions). The study did not meet the primary endpoint (1-year OS), even if it was able to delay thoracic progression in the RT group [30]. The phase III CREST trial randomized ES-SCLC with any response to 4–6 cycles of chemotherapy into a ctRT group (30 Gy in 10 fractions) compared to the observation one. The results showed a 2-year OS in favor of ctRT, 13% vs. 3% (*p* = 0.004), and also the PFS was improved in the ctRT group HR: 0.73 (95% CI 0.61–0.87; *p* = 0.001) without determining an excess of toxicities [31]. More recently, the Italian TRENDS trial confirmed that ctRT reduces the risk of intrathoracic progression, being also well tolerated [32]. Even the role of ctRT is still debated, as clearly, no data can be drawn from the two immunotherapy trials where ctRT was not allowed. As a matter of fact, thoracic ctRT after any response to chemo-immunotherapy is not recommended in the American and European guidelines [1,29]. However, retrospective data, including patients with ES-SCLC treated with immunotherapy combined with chemotherapy with/without ctRT, demonstrated that the addition of thoracic ctRT improved PFS and local control, even if it did not improve OS [33]. On the other hand, the TREASURE study, recently presented at ESMO 2023, showed that the addition of thoracic ctRT after first-line chemo-immunotherapy can be associated with an increased rate of severe toxicities, (especially pneumonitis, sepsis and multiorgan failure), so that the recruitment was permanently discontinued [34]. The results of our survey showed that the majority of respondents (83%) propose ctRT when patients present an intra- and extrathoracic response to systemic chemo-immunotherapy. The most used schedule is 30 Gy in 10 fractions (49%), followed by the use of higher doses such as 39–45 Gy in 13–15 fractions (23%) or 60 Gy in 30 fractions (22%). Only 6% of radiation oncologists use the twice-daily regimen (45 Gy in 30 fractions BID) in this setting. The use of modern radiotherapy techniques is preferable in order to achieve a reduction in treatment-related toxicity and improve treatment tolerance. Half of respondents perform ctRT in association with immunotherapy, 36% before starting immunotherapy, and only 14% suspend immunotherapy during thoracic cRT.

Finally, the treatment of brain metastases is very challenging, as patients are often excluded from clinical trials evaluating the use of radiosurgery (RS) and SRT. The FIRE study concluded that the use of RS or SRT for the first-line treatment of brain metastases from SCLC presents similar outcomes to those shown by other histologies [35]. After the publication of these results, an American survey showed an improved use of SRT for the treatment of brain metastases from SCLC among American radiation oncologists [36]. In our survey, 39% prefer WBRT in the case of intracranial failure if PCI was not previously performed, while 43% always propose SRT independently from previous PCI. The introduction of immunotherapy revolutionized the treatment in patients with progressive disease. Recently, in the SAMOS study, data about the use of SRT for extracranial and intracranial metastases from oligoprogressive SCLC were retrospectively collected [37]. The results suggested that the use of SRT could delay further systemic therapies, prolonging the use of the same treatment. In our survey, only 30% of respondents propose a second-line systemic treatment in the case of oligoprogressive disease, while the majority of respondents (about 70%) continue immunotherapy, adding SRT to the site of oligoprogression.

Some limitations of this study must be acknowledged, such as the relatively low response rate (40%), despite the good result obtained when compared with a previous Italian national survey on the same topic [16]. This study focuses on the management of ES-SCLC, for which radiation oncologists are not typically involved in the first place. Consequently, considering that most of the specialists involved were radiation oncologists, we can hypothesize that not all of them felt confident in answering the questionnaire or had the possibility in their clinical routine to develop a strong opinion on the matter. Furthermore, the lack of personalized invitations, regular reminders and the use of a single recruitment strategy could have negatively affected the attractiveness of the survey. Likewise, the survey structure with closed-ended questions, without the possibility to collect free text options in the questions, could have lowered the response rate. Another limitation of this study is related to the fact that the answers were provided mainly by radiation oncologists, leading to a prevalence of this point of view compared to that of other specialists such as medical oncologists or pulmonologists. On the other hand, the lively debate on the use of thoracic consolidation and PCI is even more burning and controversial with the advent of immunotherapy. Therefore, the conclusions of this work must be taken with caution, as it is not a clinical trial but rather an overview of the perception of the use of radiotherapy in ES-SCLC. The value of this article lies in laying the foundations for a broader and more articulated multidisciplinary discussion aimed at identifying the best and appropriate therapeutic options from the perspective of integrated treatments in this clinical scenario.

To our knowledge, our paper represents the first survey in the immunotherapy era regarding ES-SCLC open questions in the Italian radiation oncology community, and its results may help to optimize and personalized the treatment of this aggressive disease.

## Figures and Tables

**Table 1:** Characteristics of respondents.

Medical Specialty (%)	Radiation Oncologist	89	Pneumologists	4	Medical Oncologist	6	Other Specialties	1
Years of experience (%)	<5 years	30	6–10 years	19	11–15 years	31	>15 years	20
Geographical distribution (%)	North %	51	Centre	21	South	28		
Institution of work (%)	Academic hospital	24	Non-academic hospital	46	IRCCS Cancer Care Center	24	Private hospital	6
Time spent working on lung cancer (%)	<50%	31	50–70%	44	70–90%	15	<90%	20

## Data Availability

The data presented in this study are available on request from the corresponding author.

## Supplementary Materials

The following supporting information can be downloaded at: https://www.mdpi.com/article/10.3390/curroncol31110501/s1, Table S1: Questions posed to respondents.
