# Supplementary material for: Current Radiotherapy Management of Extensive-Stage Small-Cell Lung Cancer in the Immunotherapy Era: An Italian National Survey on Behalf of the Italian Association of Radiotherapy and Clinical Oncology (AIRO)
Source: Curr Oncol. 2024 Nov 1;31(11):6791–802. doi: 10.3390/curroncol31110501 (PMC11592792; doi:10.3390/curroncol31110501)
Supplement: Supplementary file 1 [file curroncol-31-00501-s001.zip › curroncol-3255492-supplementary.pdf]

Table S1. Questions posed to respondents.

DEMOGRAPHICS AND EXPERTISE IN ED-SCLC TREATMENT

- Which is your medical specialty?
- How long have you been working as a radiation oncologist?
- What type of healthcare institution do you work for?
- What percentage of your time do you spend on lung cancer disease?
- In which italian geographical area do you practice?
- Which medical figure is usually responsible for/handle patients diagnosis and staging in your Centre?
- Do you have a MDT discussion dedicated SCLC cases in your Institution?
- How many pts with newly diagnosed SCLC came to your department in the last year?
- How many pts with newly diagnosed ES-SCLC came to your department in the last year?

MANAGEMENT OF ED-SCLC

Role of systemic treatment

- Which is your first therapeutic approach in case of pts with ED- SCLC and PS=0-1?
- What CHT regimen do you prefer in your clinical practice in case of patients with ED- SCLC and PS=0-1?
- What CHT regimen do you prefer in your clinical practice in case of patients with ED- SCLC and PS=2 (due to tumour-related symptoms)?
- Considering the combination of CHT and IT in patients with ES-SCLC, how many cycles of CHT do you usually plan in your clinical practice?

Role of PCI

- Considering the results from IT plus CHT in ES-SCLC and given the possibility of maintenance therapy, do you consider PCI in these patients in your clinical practice?
- If prescribed, when PCI is usually performed in your clinical practice?
- If prescribed, which radiation technique do you usually choose for PCI?

Role of thoracic consolidative RT

- Taking into account the results achieved by consolidation IT in ES-SCLC patients, do you consider RT for intrathoracic disease?
- Which schedule do you usually choose for thoracic RT in ES-SCLC patients?
- When do you usually start thoracic RT in ES-SCLC?

Management of oligoPD

- What treatment do you usually recommend in case of ES-SCLC patient with intracranial oligoPD during manteinance IT?
- What treatment do you usually recommend in case of ES-SCLC patient with extracranial oligoPD during manteinance IT?

pts patients, M*DT* multidisciplinary team, *SCLC* small cell lung cancer, *ES-SCLC* extended stage small cell lung cancer, *PS* performance status, *CHT* chemotherapy, *IT* immunotherapy, *PCI* prophylactic cranial irradiation, *RT* radiation therapy, *oligoPD* oligoprogression disease
